# Socioeconomic Differences in Tobacco Smoking in Italy: Is There an Interaction between Variables?

**DOI:** 10.1100/2012/286472

**Published:** 2012-03-12

**Authors:** Leda Semyonov, Gianluca Iarocci, Antonio Boccia, Giuseppe La Torre

**Affiliations:** ^1^Department of Public Health and Infectious Diseases, Sapienza University of Rome, Viale Regina Elena 324, 00181 Rome, Italy; ^2^Istituto Superiore per la Protezione e la Ricerca Ambientale (ISPRA), 00144 Rome, Italy; ^3^Eleonora Lorillard Spencer Cenci Foundation Rome, Italy

## Abstract

*Objectives*. To assess the influence of sociodemographic factors on smoking habits in Italy and if an interaction exists between these variables. *Methods*. Data from the national survey “Health Conditions and Healthcare Services Use” in 2005 were used. The independent association between tobacco smoking and sociodemographical variables was assessed using logistic regression analysis. Interactions between variables were investigated calculating the synergism index (SI). *Results*. Sample population consists of 109.829 subjects (over 15 years). 21.9% are current and 21.8% are former smokers. Current smokers are mostly 45–54-years old males, from Central Italy, unemployed, divorced or separated but having a good health status without chronic medical conditions. Ever smokers are mostly 45–54 years old males, from Northeast Italy, unemployed, with chronic conditions. People with a university degree and with a good household income have the lowest OR for both conditions. A synergistic effect was found between marital status and educational level (for ever smokers SI = 1.96; for current smokers SI = 1.67). *Conclusions*. Smoking is prevalent in lower socioeconomic groups and there is the strong need to increase social, economic and cultural capital in order to reduce it.

## 1. Introduction

Despite the preventive measures that have been implemented by various governments, tobacco smoking is still one of the major causes of avoidable illnesses and premature death in Europe [1, 2]. Certainly, smoking cessation is a dynamic process with different levels of motivation and confidence in quitting [3–5].

Some authors tried to investigate whether an effective association between smoking and socioeconomic status exists: they have demonstrated that males with a low social status have a high probability of starting to smoke and a low probability of quitting smoking [6–9]. Recent studies have investigated and proved the association between social inequalities and smoking cessation [10–12].

Other studies have identified the contextual, socio-environmental mechanisms that influence smoking behaviors and showed factors such as socioeconomic status, education level, the partner's smoking habit, and passive smoking [13, 14].

A cross-sectional study based on 3 Italian cohorts has demonstrated that 40-year-old people with a low socioeconomic status have a higher risk of smoking than people with high socioeconomic status [15]. This study's aim was to describe socioeconomic inequalities in initiation and cessation rates of smoking and the educational differences in the probability of taking up the habit, the probability of quitting, and the prevalence of smoking for each birth cohort and for both genders.

Conversely, the goals of the present study are

- to evaluate the existence of sociodemographic factors associated with and/or influencing smoking habits in the Italian population;
- to verify if an interaction does exist between sociodemographic variables in explaining these smoking habits.

## 2. Methods

### 2.1. Data Source and Study Design

This cross-sectional study was based on the national survey “Health Conditions and Healthcare Services Use”, carried out by the Italian National Centre of Statistics (ISTAT) in 2005.

In the original survey, a questionnaire was administered in March, June, September, and December 2005 in order to avoid possible seasonal effects on the answers.

The sample population of this study was extracted from the ISTAT survey considering all individuals aged 15 years and older, regardless of their health conditions.

### 2.2. Measurement of Dependent and Independent Variables

This study analyzed the association between smoking habit (dependent variable) and gender, age, educational level, Italian macroregion of residence, chronic medical conditions, occupational status, marital status, self-assessed health status, and self-assessed household income (independent variables).

Smoking habits were classified as current smokers, ever smokers, and never smokers. Current smokers category refers to actual smoking behavior, while ever smokers category was comprised by current smokers and former smokers.

The macroregions of residence (Northeast, Northwest, Centre, South, Islands) were included because of their socioeconomic differences.

The occupational status was classified as follows: employed, unemployed, or in search of first occupation, housewives or students, and other.

The marital status was described as married, single, divorced or separated, and widowed.

Concerning the educational level, it was classified, according to ISTAT categories, as primary education, lower secondary education or professional school, upper secondary education, and university degree.

A further indicator of socioeconomic status was the self-assessed household income (Scarce/absolutely insufficient and very good/adequate).

Self-assessed health status was categorized as not good/poor and very good/good; respondents were also classified as having at least one chronic medical disease or not having any.

### 2.3. Statistical Analyses

A univariate analysis was performed by using the *Chi-square* test to investigate the association between the dependent and the independent variables.

Moreover, a multiple logistic regression analysis was performed, assessing the association of smoking habit with the independent variables. The reference groups taken into account were males (gender), 15–24 years (age), primary education (educational level), residence in Northeastern macroregion (region of residence), no chronic medical conditions, employed (occupational status), married (marital status), not good/poor health status (self-assessed health status), and scarce/absolutely insufficient income (self-assessed household income).

Results are presented as odds ratios (OR) and 95% confidence intervals (95% CI).

The possible interaction between sociodemographic variables was tested using the synergism index, calculated as follows: *S* = [OR_11_ −1]/([OR_01_ + OR_10_] −2), where OR_11_ is equal to OR of the joint effect of two risk factors and OR_10_ and OR_01_ are equal to OR of each risk factor in the absence of the other. A value of* S *equal to unity was interpreted as indicative of additivity, whereas a value greater than unity was indicative of superadditivity and synergism [16, 17].

The level of statistical significance was set at *P* < 0.05.

All statistical analyses were performed by using Statistical Package for the Social Sciences, Version 15.0 (SPSS Inc., Chicago, IL, USA).

## 3. Results

### 3.1. General Population

The Italian population aged 15 and older comprises 49.769.729 individuals (source population); 109.829 subjects belonging to this age category were enrolled as part of the study population sample. In this population, 21.9% are current smokers, while 21.8% are former smokers; 56.7% are never smokers.

The participants were mainly females (51.84%), aged over 65 years (22.38%), with lower secondary education or professional school (37.65%), resident in the Northwestern macroregion (26.74%), having no chronic condition (94.51%), employed (46.70%), married (55.76%), with a self-assessed very good/good health status (93.20%), and a very good/adequate self-assessed household income (60.89%).

Table 1 shows that the prevalence of *Current Smokers* is significantly different between males (27.8%) and females (16.4%). Regarding *Former Smokers, *the prevalence is 21.8% and it is significantly different between males (29.6%) and females (14.6%).

The highest prevalence of current smokers was noticed among people aged 45–54 years (28.3%) living in Central Italy (23.8%) and the lowest prevalence was among people aged over 65 years (9%).

The age group with the highest prevalence of former smokers was the age category of 55–64 years prevalently living in Northeast of Italy (24.9%).

Most of the current smokers are unemployed or first time job-seekers (30.2%), with a lower secondary education or professional school (26.4%). Former smokers also just attended primary education.

Concerning the marital status, current smokers were mainly divorced or separated with a very good/adequate self-assessed household income (24.3%), while most of the former smokers were married (27.5%) and with scarce/absolutely insufficient household income (26.8%).

In addition, current smokers seemed to have a good health status (22.6%) and no chronic medical conditions (22%). The opposite situation emerges among former smokers: the highest prevalence was found among people with a poor health status (27%) and at least one chronic medical conditions (28.7%).

### 3.2. Current Smokers

Univariate analysis (Table 2) revealed significant differences.

The male population of 35–44 years of age, living in Central Italy, unemployed or first time job-seekers but having a very good health status with no chronic medical conditions, have a significantly higher odds of current smoking habits.

Regarding the marital status, being divorced or separated seems to be a risk factor for current smoking.

On the contrary, high levels of education, such as a university degree, act as protective factors.

The multivariate analysis (Table 2) confirmed most of the significant differences found with the univariate analysis, such as differences between gender, educational level, area of residence, having chronic medical conditions, occupational status as well as marital status and health status.

Multivariate analysis, unlike the univariate one, revealed that the age group 45–54 is more prone to smoke.

Regarding the household income, while the univariate analysis shows that having good/adequate household income acts as a risk factor, the multivariate analysis underlines the opposite situation (adjusted OR versus scarce/absolutely insufficient self-assessed household income 0.897, 95% CI 0.896–0.899).

### 3.3. Ever Smokers

Regarding *Ever Smokers*, the univariate analysis (Table 3) pointed out some significant differences.

Males of 45–54 years of age, from Central Italy, seemed to be more inclined to smoke. Also, people with a university degree and a good self-assessed household income have the lowest OR (respectively, 0.908 and 0.912).

In contrast to *current smokers*, people with chronic conditions have the highest odds of being an ever smoker (OR = 1.235).

When analyzing health status and marital status, it emerged that people with a good self-assessed health status as well as divorced or separated people have a higher OR for smoking (respectively, 1.201 and 1.667).

As far as occupational status is concerned, the univariate analysis found the highest OR for people with other conditions (OR = 1.26), while the multivariate analysis showed that unemployed people have the highest OR (adjusted OR versus employed 1.093, 95% CI 1.090–1.096).

The multivariate analysis (Table 3) confirmed all the other significant differences showed by the univariate analysis: males of 45–54 years of age, living in Central Italy, with a lower secondary education or professional school have the highest OR for smoking; the same emerges for divorced or separated people with a good health status but with chronic medical conditions and scarce self-assessed household income.

### 3.4. Synergism

Table 4 shows the synergistic interaction between sociodemographic variables in influencing ever and current smoking. There was an indication for the additivity and synergism between two risk factors: low educational level (not having a university degree) and marital status (being divorced). It clearly appears the joint effect and the synergistic interaction of these risk factors influencing both *Ever Smokers* (1.96) and *Current Smokers* (1.67).

The highest interaction (*S* = 2) was found between the variables sex (male gender) and educational level (low educational level), but for current smoking only.

## 4. Discussion

The present study highlights the differences between Italian current and ever smokers related to socioeconomic factors. This study also confirms the results of previous studies about socioeconomic inequalities in health determined by gender, age, education, geographical region, self-assessed health status, and household incomes [4, 6, 7, 15, 18, 19].

Similarly to the study carried out by Laaksonen et al. [5], we have found that all socioeconomic indicators were strongly associated with smoking in both men and women.

When considering chronic medical conditions, it emerges that current smokers seem to have a good health status and no chronic medical conditions. In contrast, ever smokers have a poor self-assessed health status: maybe this condition is due to the fact that people who smoked in the past have now developed some chronic diseases, while current smokers, having a relatively good health status, continue to smoke. This probably also explains the difference in age groups between current (45–54 years of age) and ever smokers (55–64 years of age): the difference in age can be related to the fact that former smokers have smoked in the past, so now they are older than current smokers.

One additional difference between current and ever smokers is related to the economic situation. In particular, among current smokers the highest prevalence (24.26%) is concerning people with a very good/adequate household income, while among former smokers this figure is mainly present (26.83%) in individuals with a scarce/absolutely insufficient household income.

When analyzing the geographic area of residence, it appears that current smokers are more often from Central Italy, while former ones are from the Northeast of Italy. A possible explanation of the latter figure, could derive from the fact that Northeastern area represents the richest macroregion in Italy.

Looking at occupational status and educational level, it can be underlined that low socioeconomic factors are strictly linked to smoking habits: both current and former smokers are mostly unemployed, or first time job-seekers, and with a low educational level.

Concerning marital status, current smokers are mostly divorced or separated (34,41%): this is probably linked to the fact that stress factors like marital problems seem to act as an encouragement to smoke. Most former smokers are married (27.51%).

Concerning the synergistic interaction between sociodemographic variables in influencing ever and current smoking, as suggested by Abel [20], we were able to develop models showing that the interaction between the three forms of capital can play an important role in the unequal distribution of health. In fact, educational level, gender and marital status seem to interact with each other, confirming how cultural, social and economic capital are key processes in determining health lifestyles and thus health inequalities.

The strength of our study, analyzing a large database considering more than 100.000 individuals, is the demonstration that socioeconomic factors are strictly related to smoking habits, even with an interaction relationship.

## 5. Conclusions

In conclusion, smoking habits are more prevalent in lower socioeconomic groups. Public health should attempt to adopt antismoking measures as well as information campaigns in order to reduce smoking among the socioeconomically disadvantaged classes (people with lower education, unemployed) [18, 19, 21, 22].

Antismoking campaigns must be implemented in schools, and in these settings these preventive interventions must be put in place before children start experimenting tobacco, in order to influence smoking attitudes and behavior [23, 24].

Moreover, prevention interventions must be accompanied by mass media campaign and by GPs activities with the aim of sensitizing especially young people and the whole lower socioeconomic class to tobacco smoking.

## Figures and Tables

**Table 1:** Socioeconomic and demographic characteristics of participants, year 2005.

		Current	Former	No smokers	Total
Total		21,92	21,84	56,24	100

Gender	Male	27,84	29,57	42,59	48,16
	Female	16,43	14,64	68,93	51,84

Age (years)	15–24	21,39	5,79	72,82	12,18
	25–34	27,82	14,84	57,34	17,06
	35–44	28,07	19,59	52,34	18,89
	45–54	28,28	26,32	45,4	15,3
	55–64	20,65	29,19	50,16	14,2
	65+	8,99	30,05	60,96	22,38

Educational level	University degree	20,14	21,51	58,35	9,26
	Upper secondary education	23,59	20,86	55,55	26,14
	Lower secondary education or professional school	26,45	20,77	52,78	37,65
	Primary education	14,6	24,37	61,03	26,95

Italian macroregion of residence	North-west	22,19	23,25	54,56	26,74
	North-east	21,38	24,95	53,67	19,01
	Centre	23,76	23,54	52,7	19,45
	South	20,77	17,6	61,63	23,56
	Islands	21,44	19,11	59,45	11,24

Chronic medical conditions	None	22,03	21,44	56,53	94,51
	One at least	20,03	28,68	51,29	5,49

Occupational status	Employed	29,37	22,3	48,33	46,7
	Unemployed, in search of first occupation	30,24	13,77	55,99	5,56
	Housewife, student	13,92	11,14	74,94	25,18
	Other	13,39	34,79	51,82	22,56

Marital status	Single	25,62	12	62,38	29,63
	Married	20,69	27,51	51,8	55,76
	Divorced, separated	34,41	21,36	44,23	5,38
	Widowed	10,21	19,37	70,42	9,22

Self-assessed health status	Very good/good	22,61	21,45	55,94	93,2
	Not good/poor	12,56	27,05	60,39	6,8

Self-assessed household income	Very good/adequate	24,26	18,62	57,12	60,89
	Scarce/absolutely insufficient	18,28	26,83	54,89	39,11

**Table 2:** Sociodemographic predictors of *Current smoking*—Multiple logistic regression model, year 2005.

		Crude OR (95% CI)	Adjusted OR (95% CI)	
Gender	Male	1	1	Reference
	Female	0,51	0,598	0,597–0,599

Age (years)	15–24	1	1	Reference
	25–34	1,475	1,304	1,301*–*1,308
	35–44	1,514	1,33	1,326*–*1,334
	45–54	1,503	1,396	1,392*–*1,401
	55–64	0,915	1,052	1,048*–*1,056
	65+	0,286	0,452	0,450*–*0,454

Educational level	Primary education	1	1	Reference
	Lower secondary education or professional school	1,515	1,145	1,142*–*1,147
	Upper secondary education	1,138	0,901	0,898*–*0,903
	University degree	0,889	0,688	0,686*–*0,690

Italian macroregion of residence	Northeast	1	1	Reference
	Northwest	1,022	1,063	1,061*–*1,065
	Centre	1,139	1,204	1,201*–*1,206
	South	0,914	0,996	0,993*–*0,998
	Islands	0,968	1,04	1,037*–*1,043

Chronic medical conditions	None	1	1	Reference
	One at least	0,886	0,986	0,983*–*0,989

Occupational status	Employed	1	1	Reference
	Unemployed, in search of first occupation	1,589	1,083	1,079*–*1,086
	Housewife, student	0,495	0,607	0,606*–*0,608
	Other	0,479	0,736	0,734*–*0,738

Marital status	Married	1	1	Reference
	Divorced, separated	1,948	1,774	1,769*–*1,779
	Widowed	0,378	1,086	1,082*–*1,090
	Single	1,347	1,227	1,224*–*1,229

Self-assessed health status	Not good/poor	1	1	Reference
	Very good/good	2,034	1,172	1,168*–*1,177

Self-assessed household income	Scarce/absolutely insufficient	1	1	Reference
	Very good/adeguate	1,432	0,897	0,896*–*0,899

analysis [crude odds ratio (OR)] and multivariate analysis (adjusted OR). Dependent variable: “being a current smoker.”

**Table 3:** Sociodemographic predictors of* Ever smoking status*—Multiple logistic regression model, year 2005.

		Crude OR (95% CI)	Adjusted OR (95% CI)	
Gender	Male	1	1	Reference
	Female	0,334	0,387	0,386–0,387

Age (years)	15–24	1	1	Reference
	25–34	0,947	1,587	1,583–1,592
	35–44	1,215	1,761	1,756*–*1,766
	45–54	1,675	2,442	2,435*–*2,449
	55–64	1,331	2,132	2,125*–*2,139
	65+	0,779	1,545	0,450*–*0,454

Educational level	Primary education	1	1	Reference
	Lower secondary education or professional school	1,252	1,368	1,366*–*1,371
	Upper secondary education	1,039	1,237	1,235*–*1,240
	University degree	0,908	0,935	0,933*–*0,937

Italian macroregion of residence	Northeast	1	1	Reference
	Northwest	1,096	0,96	0,959–0,962
	Centre	1,195	1,068	1,066–1,070
	South	0,748	0,762	0,761–0,764
	Islands	0,861	0,843	0,841–0,844

Chronic medical conditions	None	1	1	Reference
	One at least	1,235	1,265	1,261*–*1,268

Occupational status	Employed	1	1	Reference
	Unemployed, in search of first occupation	1,011	1,093	1,090*–*1,096
	Housewife, student	0,334	0,591	0,590–0,592
	Other	1,26	0,997	0,995–0,999

Marital status	Married	1	1	Reference
	Divorced, separated	1,667	1,297	1,293*–*1,300
	Widowed	0,509	0,732	0,730–0,734
	Single	0,698	0,82	0,819–0,822

Self-assessed health status	Not good/poor	1	1	Reference
	Very good/good	1,201	1,102	1,099–1,105

Self-assessed household income	Scarce/absolutely insufficient	1	1	Reference
	Very good/adequate	0,912	0,812	0,811–0,813

analysis [crude odds ratio (OR)] and multivariate analysis (adjusted OR). Dependent variable: “being a current or a former smoker.”

**Table 4:** Synergistic interaction between Sociodemographic variables in influencing ever and current smoking.

Variables		Ever smokers AOR (95% CI)*	Synergistic interaction	Current smokers AOR (95% CI)*	Synergistic interaction
No university degree	Divorced				
No	No (reference)	1		1	
Yes	No	1.08	1.96	1.08	1.67
No	Yes	1.44		1.44	
Yes	Yes	2.02		2.02	

No university degree	Males				
No	No (reference)	1		1	
Yes	No	0.75	−0.88	0.75	−1.33
No	Yes	0.65		0.65	
Yes	Yes	1.53		1.53	

No university degree	Low income				
No	No (reference)	1		1	
Yes	No	1.41	0.5	1.24	0.62
No	Yes	1.13		1.21	
Yes	Yes	1.27		1.28	

Males	North-Centre				
No	No (reference)	1		1	
Yes	No	1.41	0.24	1.03	−0.06
No	Yes	1.44		1.44	
Yes	Yes	1.27		0.97	

Males	Divorced				
No	No (reference)	1		1	
Yes	No	0.74	0.36	0.56	0.82
No	Yes	2.23		2.41	
Yes	Yes	1.35		1.80	

Females	Divorced				
No	No (reference)	1		1	
Yes	No	0.45	−1.52	0.42	−1.38
No	Yes	0.74		0.56	
Yes	Yes	2.23		2.41	

Males	Low income				
No	No (reference)	1		1	
Yes	No	0.58	−1.07	0.96	2.00
No	Yes	0.74		1.06	
Yes	Yes	1.73		1.04	

*AOR: odds ratio adjusted for age and gender.
